# Clavicular hook plate may induce subacromial shoulder impingement and rotator cuff lesion - dynamic sonographic evaluation

**DOI:** 10.1186/1749-799X-9-6

**Published:** 2014-02-06

**Authors:** Hsin-Yu Lin, Poo-Kuang Wong, Wei-Pin Ho, Tai-Yuan Chuang, Yi-Shyan Liao, Chin-Chean Wong

**Affiliations:** 1 Department of Orthopaedic Surgery, Wan Fang Hospital, Taipei Medical University, No. 111, Sec. 3, Xinglong Road, Taipei 11696, Taiwan; 2 Graduate Institute of Clinical Medicine, College of Medicine, National Taiwan University, Taipei 10617, Taiwan; 3 Department of Orthopaedic Surgery, Lin Shin Hospital, Taichung 408, Taiwan

**Keywords:** Hook plate, Subacromial impingement, Rotator cuff

## Abstract

**Background:**

Clavicular hook plates are effective fixation devices for distal clavicle fractures and severe acromioclavicular joint dislocations. However, increasing number of studies has revealed that subacromial portion of the hook may induce acromial bony erosion, shoulder impingement, or even rotator cuff damage. By sonographic evaluation, we thus intended to determine whether the presence of hook plate may induce subacromial shoulder impingement and its relationship relative to surrounding subacromial structures.

**Methods:**

We prospectively followed 40 patients with either distal clavicle fracture or acromioclavicular joint dislocation that had surgery using the *Arbeitsgemeinschaft für Osteosynthesefragen* (AO) clavicular hook plate. All patients were evaluated by monthly clinical and radiographic examinations. Static and dynamic musculoskeletal sonography examinations were performed at final follow-up before implant removal. Clinical results for pain, shoulder function, and range of motion were evaluated using Constant-Murley and Disability of Arm, Shoulder, and Hand (DASH) scores.

**Results:**

Clinically, 15 out of 40 patients (37.5%) presented with subacromial impingement syndrome and their functional scores were poorer than the non-impinged patients. Among them, six patients were noted to have rotator cuff lesion. Acromial erosion caused by hook pressure developed in 20 patients (50%).

**Conclusions:**

We demonstrated by musculoskeletal sonography that clavicular hook plate caused subacromial shoulder impingement and rotator cuff lesion. The data also suggest an association between hardware-induced impingement and poorer functional scores. To our knowledge, the only solution is removal of the implant after bony consolidation/ligamentous healing has taken place. Thus, we advocate the removal of the implant as soon as bony union and/or ligamentous healing is achieved.

## Background

The surgical methods for unstable distal clavicle fractures (Neer type II, III) and acromioclavicular (AC) joint dislocation (Rockwood type III) share many similarities. Conventional methods utilizing extraarticular or transarticular Kirshner wire [1-3], Knowles pin [4,5], tension bands [6], and coracoclavicular screws [7], although simple, often carry considerable risk for complications [5-10]. These include uncontrollable pin migration, pin breakage, loss of fixation, and non-union [8-12].

The clavicular hook plate is designed to fit anatomically to the acromion and clavicle, with the hook extending from the plate acting as a lever beneath the acromion [13]. Anatomical configuration of the clavicle and acromion is maintained when the plate is properly placed along the clavicle and fixed with screws. Several studies using the hook plate in treating these fractures and dislocations have shown satisfactory clinical results as defined by reliable fixation, fast bony union and/or ligamentous healing, and few complications [1,14-21].

Some clinicians consider it safe to retain the hardware, but most authors advocate early removal of the plate as soon as bony union and/or ligamentous healing is achieved [4,13,18,20]. Several studies have noted that excessive hook pressure may lead to subacromial bony erosion and acromial osteolysis [13,18,20]. However, the main concern is that the plate may cause subacromial shoulder impingement or even rotator cuff tear [13,18,20,22]. Ikuta et al. reported that 5 out of 47 patients with AC dislocation or distal clavicle fracture treated with a Wolter plate, a type of clavicular hook plate, developed shoulder impingement syndrome [23]. Physical examination and conventional radiographic modalities are sensitive but not very specific, so a diagnosis based on these examination results alone is not always accurate [24-26]. MRI is a reliable technique to evaluate shoulder abnormalities, but it provides only static and indirect evaluation of the shoulder [27-29]. In contrast, musculoskeletal sonography can characterize a spectrum of abnormalities of impingement syndrome by providing real-time, dynamic, and reliable information [30-35].

In this study, we sought to determine whether the clavicular hook plate fixation may induce subacromial shoulder impingement by dynamic musculoskeletal sonography as an evaluation tool. Moreover, we intended to know the association between the hardware-induced problems and clinical outcome of patients in terms of shoulder functional score.

## Methods

### Subjects

We prospectively followed all 42 patients (32 men, 10 women) with 32 unstable distal clavicle fractures (Neer type II, III) and 10 AC joint dislocation (Rockwood type III) treated with clavicular hook plate (Synthes® medical company, Bettlach, Switzerland) from December 2007 to January 2010. All patients were informed that the hook plate was to be removed after bony union and/or ligamentous healing was achieved on radiographs and should not be retained for longer than 6 months. During follow-up, one female elderly patient (82 years old) who died of unrelated causes and another patient with a pre-existing neurological deficit on the injured arm were excluded. These left 40 patients with a minimum follow-up of 12 months (mean, 13.6 months; range, 12–17.2 months). No patients were lost to follow-up. All patients enrolled in our study had non-pathological fractures, no previous rotator cuff lesions, and normal shoulder function before injury. None of the 40 patients had previous trauma (fracture or dislocation) or surgery on the affected shoulder. The mean age of the patients at surgery was 37.68 years. Thirty-four patients had injuries resulting from motorcycle accidents or bicycle falls, while six patients suffered the injury from a fall from a height (Table 1). All protocols were approved by the institutional review board of Wan Fang Hospital, Taipei Medical University (approval no. 98086).

**Table 1:** Demographic data of patients receiving hook plate fixation

**Characteristics**	**Results**
Mean age (years)	37.68 ± 12.09
Gender (male/female, *N* = 40)	30/10
Diabetes mellitus	1
Renal disease	0
Fall from a height	3
Bicycle/motorcycle accident	22
Mean time to surgery (days)^a^	1.52 ± 1.12
Mean time to removal of hardware (months)^a^	5.78 ± 0.83
Average follow-up (months)^a^	12.02 ± 2.38

^a^Data represent the mean ± SD.

### Surgical procedures

The hook plate was a modified stainless steel, curved 3.5 mm dynamic compression plate with a hook-like structure extending from the lateral end. The hook has two different depths (15 and 18 mm) to accommodate different thicknesses of the acromion process. Two different plate lengths with six or eight holes are available. The hook was designed to precisely engage the posterior and medial aspect of the acromion and acts as a lever to maintain the anatomical configuration of the acromion and clavicle.

The operations were performed by one of five senior orthopedic surgeons (CCW, WPH, YSL, PKW, TYC) following the method and procedure proposed by the manufacturer [36]. The operation was performed with the patients under general anesthesia and in the standard beach chair position. An incision in line with the clavicle was made, and the fracture site as well as the AC joint was identified. The fracture or dislocation was examined and reduced. The depth of the acromion was determined using a depth gauge, and the depth of the hook was decided according to the depth of the acromion. Then, the hook of the plate was passed under the acromion posterior to the AC joint. After the fracture or dislocation was reduced, the plate was placed along the length of the clavicle and fixed with screws. Taking the fracture or dislocation pattern into consideration, the plate was bent if it could not precisely fit the contour of the bone.

### Postoperative care and follow-up

Passive shoulder exercises were started 2 days postoperatively with the aid of the uninjured arm. Patients were told to use a sling for 1 month and could start active range of motion exercise thereafter. Patients were followed up every month for the first 6 months and every 3 months thereafter. AP shoulder radiographs were used for radiological assessment. Postoperative conditions such as wound infection, surgical revision, loss of implant fixation, shoulder range of motion (ROM) and radiographic evaluation for bony union and/or ligamentous healing were documented by the operating surgeon. Clinical union was defined as no tenderness (visual analog score <2) at the fracture or dislocation site. All patients had their plates removed at a mean time of 5.78 months (range 4–7 months). In patients with AC dislocation, we advised removing the plate at 3–6 months postoperatively, and patients with distal clavicle fractures were told to remove their plate at least 6 months after hardware fixation. At the final visit before the removal of the implant, all patients were examined for both active and passive shoulder ROM. A clinical diagnosis of subacromial impingement was established by a positive Neer's impingement sign. The Constant-Murley shoulder score and Disability of Arm, Shoulder and Hand (DASH) score (questionnaire in traditional Chinese version) were used for global functional assessment [37].

### Musculoskeletal sonographic evaluation

Musculoskeletal sonography was arranged and was performed by an orthopedic surgeon specialized in the field with more than 10 years of clinical experience (PKW), using HP 21376A 5–10 MHz high-resolution linear transducer on a HP ImagePoint (Hx) System (Andover, MA, USA). All patients sat on a stool with adequate exposure of the shoulder to permit easy access to both anterior and posterior aspects. The biceps tendon, acromioclavicular joint, subscapularis tendon, supraspinatus tendon, and infraspinatus tendon were examined following standard protocol. Dynamic sonography was then performed. Here, the probe was positioned in the coronal plane along the long axis of the supraspinatus tendon between the acromion and the greater tuberosity of the humerus. Then, the patient's arm was gently elevated passively by the examiner halfway between flexion and abduction with the hand pronated and the elbow in full extension. All patients were asked whether or not the movement was painful, with cessation of movement and recording of the degree of movement when the patient reported intolerable pain. The relationships between the acromion, the humeral head, and the intervening soft tissues such as the subacromial bursa and supraspinatus tendon were assessed during passive shoulder motion. All dynamic sonography examinations were recorded using a digital video camera. If the humeral head passed easily and freely underneath the acromion during shoulder motion, it was defined as a sign of no impingement. Soft tissue impingement was presumed present when (1) pooling of fluid in the lateral aspect of the subacromial/subdeltoid bursa occurred or (2) when alteration of the normally convex surface of the subacromial bursa alone or of the subacromial bursa and of the supraspinatus tendon occurred when the greater tuberosity of the humeral head passed underneath the acromion [31]. The sonography examiner (PKW) used the original grading system proposed by Bureau et al. to characterize the degree of subacromial impingement on dynamic sonography. These patients were asked to return a month later for re-evaluation of shoulder range of motion and sonographic examination after hardware removal [31].

### Statistical analysis

The Student's *t* test was used to compare the two groups. The statistic software SPSS package ą version 17.0 for Windows (SPSS, Inc., Chicago, IL, USA) was used to analyze the data; *p* values below 0.05 were considered significant.

## Results

At final follow-up, except one patient had delayed fracture union, the remaining 39 patients (97.5%) achieved clinical and radiological union and/or ligamentous healing (Table 2). No wound breakdown or infection occurred in any of the patients. The delayed union resulted from the implant fixation failure. Radiological assessment revealed cutout of the two screws from the clavicle and that the hook of the plate had partially disengaged from the acromion. The patient’s shoulder was immobilized in a sling for 1 month. The implant was removed 4 months following injury.

**Table 2:** Clinical outcome of patients receiving hook plate fixation

**Clinical outcomes**	**Results**
Union rate (%)	39/40 (97.5%)
Surgical revision (%)	0 (0%)
Wound infection (%)	0 (0%)
Loss of implant fixation (%)	1/40 (2.5%)
Acromial erosion (%)	20/40 (50%)
Rotator cuff lesion (%)	6/40 (15%)
Shoulder impingement (%)	15/40 (37.5%)
Abduction <90° (%)	15/40 (37.5%)
Forward flexion <90° (%)	10/40 (25%)

**Figure 1:**
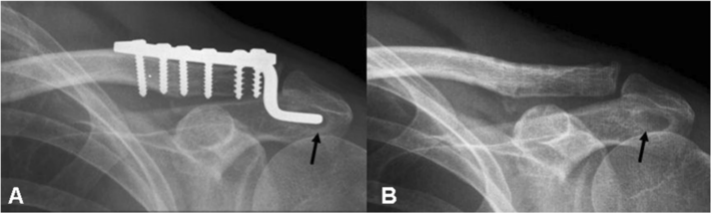
**Acromial erosion.** A 41-year-old man who received hook plate fixation for left AC dislocation. **(A)** Anteroposterior view of the left shoulder 3 months postoperatively before implant removal showed remarkable acromial osteolysis (arrow). **(B)** The bony defect was still visible 1 month after implant removal (arrow).

**Figure 2:**
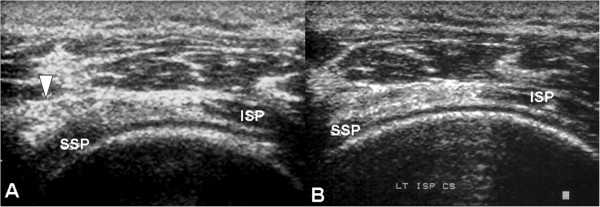
**Supraspinatus tendon attrition before and after implants removal.** Sonographic findings of a 40-year-old man who had shoulder impingement syndrome before implant removal. **(A)** A partial thickness tear was noted at the bursal aspect of the supraspinatus tendon (SSP), which resulted from repetitive mechanical attrition by the hook of the plate (arrow). The infraspinatus tendon (ISP) was intact. **(B)** 1 month after implant removal, the same patient was re-evaluated. Musculoskeletal sonography showed the dimpling lesion had become less obvious than before.

The radiographs of 20 patients (50%) demonstrated variable degrees of acromial erosion. From serial radiographic analyses, we noticed that this bony osteolysis appeared 2 months postoperatively and were still visible 4 weeks after plate removal (Figure 1). Rotator cuff lesions at the bursal aspect were noted in six patients on the operated shoulder. Under musculoskeletal sonography, mechanical cuff attrition was observed as a flattened, concave discontinuity of tendon fiber with decreased echogenicity and this finding was less remarkable after implants removal (Figure 2). These focal changes of the rotator cuff involved the posterior third of supraspinatus tendon. All six patients with rotator cuff pathology also developed subacromial shoulder impingement. No full-thickness rotator cuff tear was noted.

Of the 40 patients, 15 developed subacromial shoulder impingement. All of the subacromial impingements occurred unilaterally and specifically on the injured shoulder. Eight of the 15 patients had their dominant hand involved.

**Figure 3:**
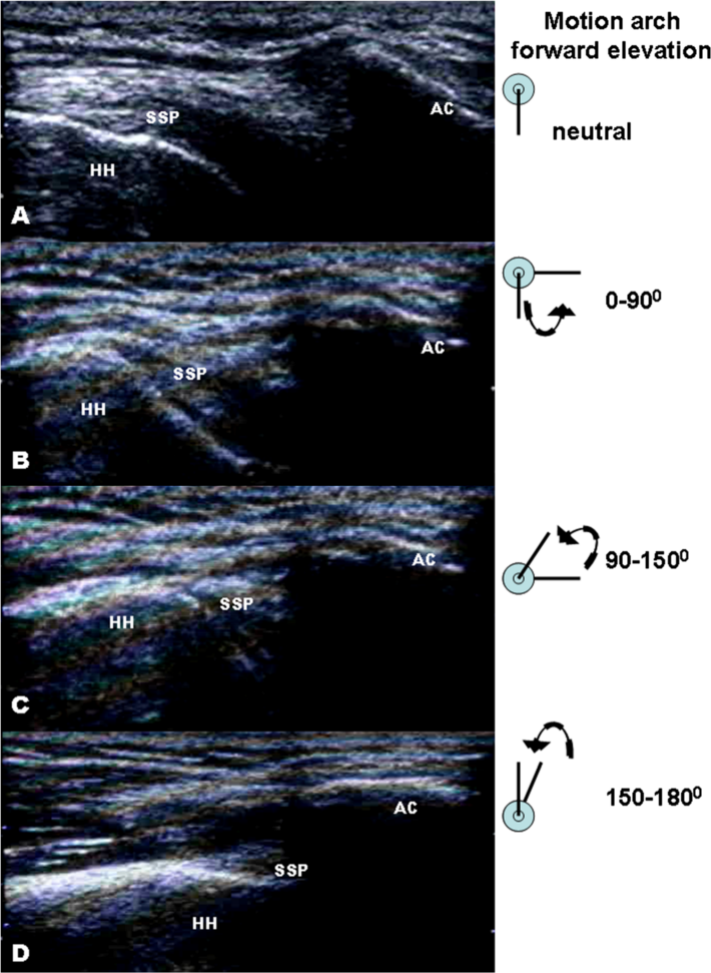
**Sonographic findings without shoulder impingement.** Dynamic musculoskeletal sonography of a 26-year-old man presented with no clinical impingement sign before implant removal. **(A–D)** Smooth passage of the supraspinatus (SSP) tendon under the acromion was observed while the shoulder was passively elevated from neutral to 180° of forward elevation. HH, humeral head; AC, acromioclavicular joint.

**Figure 4:**
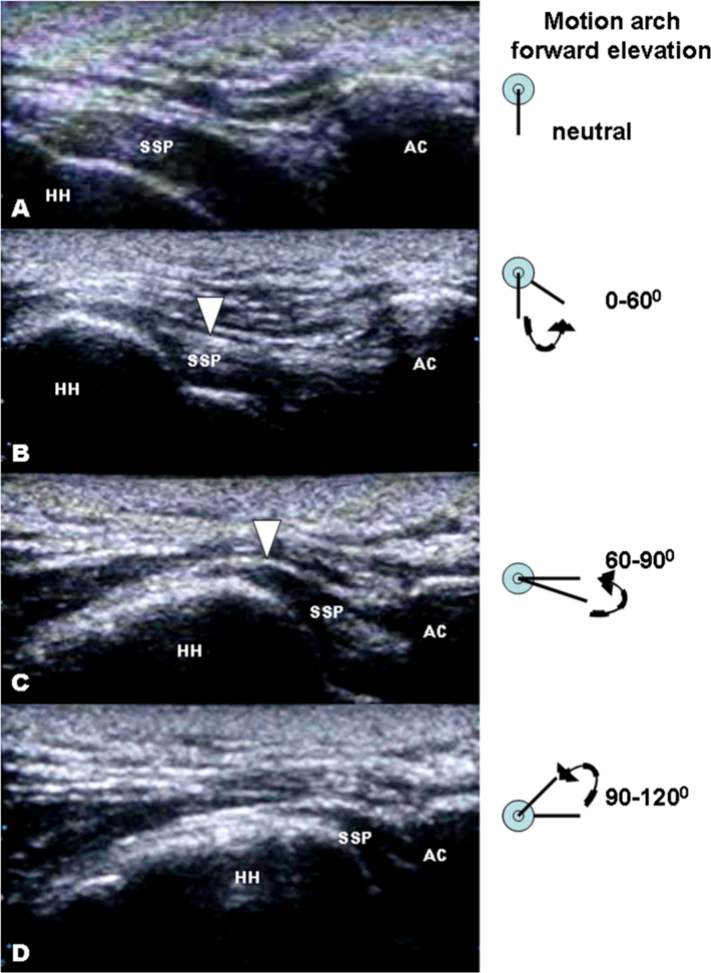
**Sonographic findings with shoulder impingement.** Dynamic musculoskeletal sonography of a 36-year-old man developed shoulder impingement syndrome after receiving hook plate fixation of a left distal clavicle fracture. The treated shoulder was passively and forwardly elevated from a neutral position towards 180° elevation. It was stopped at 120° when the patient reported intolerable pain. **(A–C)** At 90° forward elevations, bunching of supraspinatus tendon fibers (arrowheads) was noted accompanied with distention of the subacromial/subdeltoid bursa (arrow) signifying flowing fluid of bursitis. **(D)** Another remarkable finding is abnormal superior translation of humeral head with regard to the acromion obstructing its passage beneath the acromion. SSP, supraspinatus tendon; HH, humeral head; AC, acromioclavicular joint.

The mean Constant-Murley score was 83 (range 64–100) for all 40 patients. The mean DASH score was 14.43 (range 0–57). However, major differences in functional outcome existed among two groups of patients (with or without subacromial impingement). The non-impinged patients had significant higher Constant-Murley score (90.6 points; maximum score, 100 points) than patients with clinical impingement (70.2 points) with *p* value 0.001. Additionally, the non-impinged patients had less postoperative disability with mean DASH score 9.96 points compared to 18.9 points in those patients with subacromial impingement (*p* = 0.0038). The functional scores determined that the non-impinged patients had better functional recovery with less postoperative pain and better shoulder range of motion. In the dynamic sonographic examinations of those 18 asymptomatic patients without clinical subacromial impingement, no subacromial/subdeltoid (SASD) flowing fluid or bursal distention was found. Moreover, the humeral head passed freely underneath the acromion while the shoulder was elevated from neutral to 180° forward elevation (Figure 3). In the group of patients with a clinical diagnosis of subacromial impingement, three (43%) patients demonstrated evidence of subacromial bursitis (grade 2). Abnormal upward migration and difficult passage of the humeral head underneath the acromion (grade 3) were noted in four patients (Figure 4).

**Figure 5:**
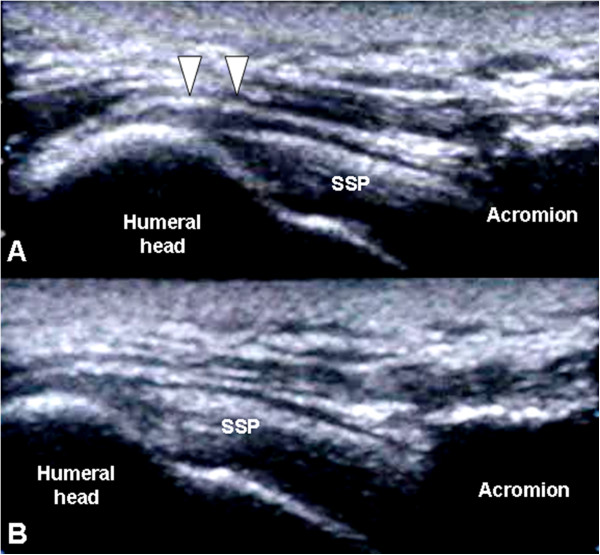
**Subdeltoid fluid and impingement before and after implants removal.** A 31-year-old man who received hook plate fixation of a right distal clavicle fracture developed shoulder impingement syndrome. **(A)** Musculoskeletal sonography revealed subacromial/subdeltoid (SASD) bursitis with flowing fluid before implant removal (arrowhead). **(B)** Four weeks after implant removal, the same patient was reevaluated. Sonographic finding showed unobstructed passage of the humeral head into the acromion and disappearance of the flowing fluid. SSP, supraspinatus tendon.

At the 1-month visit after removal of the implants, functional scores of these patients improved. The mean Constant-Murley score increased from 73 to 88 signifying marked clinical improvement, particularly in terms of active shoulder ROM. In dynamic sonographic examinations, three patients had their sonographic impingement grading lowered from grade 2 to grade 1 and four patients from grade 3 to grade 2 (Figure 5).

## Discussion

In the past few years, a number of studies have revealed that the clavicular hook plate is an effective fixation implant for distal clavicle fracture or AC dislocation regarding its reliable fixation and fast bony union [1,2,13-21]. Despite the mechanical stability as its primary advantage, some recent studies reported on the use of clavicular hook plates which have identified the subacromial impingement as one of the most notable disadvantage that causes pain and impaired function of the shoulder girdle and upper limb. The impingement rate vary considerably in different studies and can range from approximately 5% to 68% (Table 3) [13,18,20]. Patients with symptomatic impingement suffered from scraping feeling while moving the shoulder or failed to elevate or abduct their arms above 90°. Because the plate is fixed on the clavicle superiorly and the hook of the plate was inserted posterior to the AC joint, there is an underlying assumption that the hooked portion of the plate may predispose to subacromial impingement [13,18,20]. However, none of the literature that we reviewed on the use of clavicular hook plates provides evidence about the implication of the role of hardware and its possible impact on subacromial structures. In this study, we attempted to conduct a thorough and systematic analysis to answer two important questions: first, whether the hooked portion of the plate may induce subacromial impingement or even subacromial structures damage, and second, whether this hardware-induced problem would affect patients’ functional score.

**Table 3:** Surgical implant related subacromial shoulder impingement reported in hook plate studies

**Study**	**Study design**	**Case no.**	**Type of hook plate**	**Age**	**Sex ratio**	**Time of hardware retention**	**Impingement rate**	**Duration of follow-up**
Meda et al.	Case series	31	Synthes AO plate (Stratec)	49 years (25–86)	M:F; 24:7	5.56 months	6/31 (19.4%)	40 months (18–68)
Kashii et al.	Case series	34	AC hook plate (Tokyo, Japan)	40 years (21–74)	M:F; 28:6	5.3 months	2/34 (5.9%)	12.4 months (12–15)
Renger et al.	Case series	44	Synthes AO plate (B.V., Zeist)	38.4 years (18–66)	M:F; 29:15	8.4 months	33/44 (75%)	27.4 months (13–48)
ElMaraghy et al.	Cadaveric studies	15	Synthes AO plate (Paoli, PA, USA)	NA	M:F; 7:8	NA	9/15 (60%)	NA

This study has some limitations. First, the number of patient was small. Second, although those patients with sonography-diagnosed shoulder pathology denied any shoulder pain or disability before trauma, the cause-and-effect relationship of hook impingement to subacromial pathology could not be established with direct evidence. The possible effect of trauma or degeneration in rotator cuff attrition could not be totally excluded.

Classical subacromial impingement has primarily been attributed to irritation of the supraspinatus tendon by the anterior-inferior quadrant of the acromion or coracoacromial ligament [38,39]. During surgical implantation of the clavicle hook plate, it was assumed that the hooked portion of the plate is inserted posterior to the AC joint to avoid direct contact to the subacromial structures that might result in rotator cuff impingement with arm movement [36]. However, ElMaraghy et al. in their cadaveric studies reported that the 'posterior hook implantation angle’ varied widely among individuals and the angle of the hook was dictated by the unique anatomical position of each individual's clavicle relative to the acromion [40]. In this study, acromial erosion around the hook tip presented in half of the patients but has less remarkable correlation with clinical symptoms. Fifteen of our 40 patients developed subacromial impingement before hardware removal. These patients had signs of impingement and a positive Neer's sign. The clinical diagnosis of subacromial impingement was further confirmed on dynamic musculoskeletal sonography. In all seven patients, shoulder pain decreased and ROM increased after implant removal. According to our data, there was no significant difference in the subacromial impingement rate with respect to the pre-injury shoulder ROM, injury mechanism, surgical method, or duration of hardware retention. The only major difference is the mean age of patients with impingement (47.4 years), 13.6 years older than the non-impinged patients (*p* = 0.0298). These findings suggest that degenerative age-related changes of local bony as well as soft tissue structures could be a major contributing factor to the development of subacromial impingement. Our findings confirm other retrospective studies, which revealed similar findings in which older patients were reported to have more limited ROM before hardware removal [18,20]. In our study, three patients had partial thickness rotator cuff lesions at the posterior third of the supraspinatus tendon. Because the hook was inserted and engaged at the posterior aspect of the acromion, it likely impinged against the subacromial structures, such as the subacromial bursa, the rotator cuff, and even the greater tuberosity of the humerus during shoulder elevation. This highlights the importance of preventing subacromial impingement by the hook in the subacromial space. Although the manufacture's guide has mentioned the verification of full shoulder motion and exclusion of impingement before final implant fixation. Intraoperatively, it is difficult to evaluate the condition of subacromial soft tissue irritation or impingement.

From biomechanical point of view, the hook plate can provide more resistance to the deforming force of the shoulder musculatures than conventional fixation method such as tension band wire [41,42]. Moreover, the rotational movement of AC joint during shoulder abduction and flexion remains untouched. Direct and functional postoperative aftercare in patient receiving hook plate fixation is possible without marked restriction in shoulder range of motion. Patients were thus expected to have significantly better functional scores and greater ability to return to their previous level of activity. However, Meda et al. and Renger et al. reported a 19% and 68%, respectively, of shoulder impingement rates in their series of patients [18,20]. In that group of patients with subacromial impingement, they demonstrated lower clinical satisfaction, poorer functional score, and longer rehabilitation process than those in the non-impinged patients. In our study, the clinical results are consistent with the data from previous studies with nearly one third of patients presented with implant-related shoulder impingement. Unlike previous reports, we further divided the patients into two groups, those with and those without subacromial impingement, before final evaluation of functional outcome. In patients without impingement, the mean Constant-Murley score was 90.6 points and the mean DASH score was 9.96 points. There was no occurrence of rotator cuff lesion in this group. A mean Constant-Murley score of 70.2 points and mean DASH score of 18.9 were reported for the seven patients who developed subacromial impingement. There were three occurrences of rotator cuff lesion, three subacromial bursitis, and four humeral head upward migration in this group of patients. There are many causes that may induce these findings, such as humeral head upward migration which may have resulted from scapular dyskinesis or rotator cuff lesion which may have resulted from degeneration process. However, the great improvement of clinical symptoms and sonographic findings after implants removal represented the closed relationship between the hook plate and the pathology findings. The results indicated that subacromial effects of the implants may influence the clinical outcome of patients receiving hook plate fixation. Moreover, hook placement can be seen as a cause of secondary impingement through its high clinical correlation with the development of a spectrum of shoulder pathology, including subacromial bursitis, and rotator cuff lesion. Because of the highly variation of acromial anatomy [40], the prediction or determination of hook impingement is difficult during surgical procedure. Although the underlying causes of these conditions have been generally recognized as multifactorial, the interplay between additional extrinsic compression (hook placement in the subacromial space) and pre-existing degenerative age-related changes of the local bony and soft tissue structures seems to contribute variably to the formation of these shoulder pathologies. To avoid these unfavorable complications that will result in poorer functional score, it is important to consider several salient points. First, the hook should be placed at the posterior aspect of AC and securely engaged the bony part of the acromion. Second, proper selection of hook depth should be made because excessive stress is concentrated at the hook tip on the acromion causing acromial erosion if insufficient hook depth was chosen. Third, during the operation, shoulder motion, particularly abduction and forward elevation, should be verified to ensure no immediate hook impingement that will increase the risk of subacromial impingement or rotator cuff damage. Fourth, great care should be taken to the application of the hook plate in aging patients with pre-existing shoulder pathology. Finally, it is best to remove the implant as soon as bony union is achieved.

## Conclusion

In conclusion, we believe that the clavicular hook plate is useful for treating unstable clavicle fracture or AC dislocation. However, the adverse effects of the implant imposed on subacromial structures influence the patient's final functional outcome. Careful patient selection and familiarity with the special features of implant as well as surgical technique are prerequisites for good clinical results with few complications. Musculoskeletal sonography can provide useful information regarding shoulder anatomic and functional kinematics in patients who receive clavicular hook plate fixation.

## Competing interests

The authors declare that they have no competing interests.

## Authors’ contributions

CCW designed the study. CCW, WPH, YSL, PKW, and TYC operated on the patients and performed the postoperative follow-up. PKW performed the sonographic evaluation. HYL prepared the manuscript. All authors read and approved the final manuscript.
